# High Adenosine Extracellular Levels Induce Glioblastoma Aggressive Traits Modulating the Mesenchymal Stromal Cell Secretome

**DOI:** 10.3390/ijms21207706

**Published:** 2020-10-18

**Authors:** Deborah Pietrobono, Chiara Giacomelli, Laura Marchetti, Claudia Martini, Maria Letizia Trincavelli

**Affiliations:** Department of Pharmacy, University of Pisa, 56126 Pisa, Italy; Deborah.pietrobono@farm.unipi.it (D.P.); laura.marchetti@unipi.it (L.M.); claudia.martini@unipi.it (C.M.); maria.trincavelli@unipi.it (M.L.T.)

**Keywords:** glioblastoma, adenosine, tumor microenvironment, mesenchymal stromal cells, co-culture

## Abstract

Glioblastoma is an aggressive, fast-growing brain tumor influenced by the composition of the tumor microenvironment (TME) in which mesenchymal stromal cell (MSCs) play a pivotal role. Adenosine (ADO), a purinergic signal molecule, can reach up to high micromolar concentrations in TME. The activity of specific adenosine receptor subtypes on glioma cells has been widely explored, as have the effects of MSCs on tumor progression. However, the effects of high levels of ADO on glioma aggressive traits are still unclear as is its role in cancer cells-MSC cross-talk. Herein, we first studied the role of extracellular Adenosine (ADO) on isolated human U343MG cells as a glioblastoma cellular model, finding that at high concentrations it was able to prompt the gene expression of Snail and ZEB1, which regulate the epithelial–mesenchymal transition (EMT) process, even if a complete transition was not reached. These effects were mediated by the induction of ERK1/2 phosphorylation. Additionally, ADO affected isolated bone marrow derived MSCs (BM-MSCs) by modifying the pattern of secreted inflammatory cytokines. Then, the conditioned medium (CM) of BM-MSCs stimulated with ADO and a co-culture system were used to investigate the role of extracellular ADO in GBM–MSC cross-talk. The CM promoted the increase of glioma motility and induced a partial phenotypic change of glioblastoma cells. These effects were maintained when U343MG cells and BM-MSCs were co-cultured. In conclusion, ADO may affect glioma biology directly and through the modulation of the paracrine factors released by MSCs overall promoting a more aggressive phenotype. These results point out the importance to deeply investigate the role of extracellular soluble factors in the glioma cross-talk with other cell types of the TME to better understand its pathological mechanisms.

## 1. Introduction

Glioblastoma (GBM) is the most frequent primary brain tumor, with associated poor prognosis. Despite advances in surgery and chemoradiation, GBM has an aggressive course and the survival of afflicted patients has not improved significantly in the past three decades [1]. Therefore, several efforts have been made to deeply understand the mechanisms driving GBM progression. Three main tumor features interfere with the success of the therapeutic approaches: (i) the occurrence of glioblastoma stem cells (GSCs); (ii) the tumor heterogeneity; and (iii) the microenvironment and niches [2]. Emerging evidence suggests the pivotal role of tumor microenvironment (TME) in GBM progression, immune escape, local invasion and metastasis [3]. Generally, the TME consists of tumor cells, fibroblasts, endothelial cells, microglia, glioblastoma associated macrophages (GAMs), glioblastoma associated fibroblasts (TAFs), mesenchymal stromal cell (MSCs) and inflammatory cells, as well as cytokines and chemokines secreted by tumor and stromal cells [4,5]. Recently, great attention has been paid to dissect the role and the function of MSC in GBM aggressiveness. Multipotent MSCs, recruited to the tumor bulk by several soluble factors secreted by tumor cells, play complicated roles in carcinogenesis and in the modulation of tumor development [5,6,7,8,9,10]. When MSCs arrive at the area surrounding the tumor, they may differentiate into more mature mesenchymal cells, such as TAFs [11], macrophages [12] and endothelial cells [13]. Within the TME, MSC may interact with tumor cells and secrete a large range of cytokines and growth factors that may contribute to tumor cell survival, growth, motility and immune escape [14]. Interestingly, the interaction of MSC with tumor cells modifies their trophic properties through the release of various cytokines such as CXCL1, CXCL2, CXCL12 or IL-6 and metalloproteinases (MMPs) that can degrade the extracellular matrix and promote tumor migration [15,16,17]. However, the link between MSC and GBM remains obscure, as well as whether MSCs play an active role in tumor promotion or suppression [5,18,19,20,21,22,23,24,25,26].

The TME complexity reflects the GBM heterogeneity; among the several soluble factors, adenosine (ADO), a purine nucleoside, is one of the main immunosuppressive/immunomodulatory mediators [27] that play a central role in the promotion of tumor proliferation and angiogenesis [28,29,30]. ADO acts through the activation of four receptor subtypes (A_1A_R, A_2A_AR, A_2B_AR and A_3_AR). Under physiological conditions, extracellular ADO levels oscillate in concentrations between 30 and 200 nM that could activate the high-affinity AR subtype (A_1A_R and A_2A_AR); however, during inflammation, hypoxia or tumor formation, the extracellular ADO levels increases up to 100 times its normal concentration, thus eliciting complex effects as a result of the simultaneous and balanced activation of the different receptors and of nucleoside action at intracellular level [31]. In TME, ADO is produced by the activity of ecto-nucleosidetriphosphate-diphosphohydrolase (E-NTPDase1 or CD39) and ecto-5′-nucleotidase (CD73) expressed by hematopoietic (MSCs) and cancer cells that hydrolyze the extracellular ATP. Interestingly, the high expression of CD73 correlates to a poor prognosis in GBM patients [32]. 

Emerging evidences report the prominent role of adenosinergic signaling in multiple aspects of GBM aggressiveness, including GBM growth, angiogenesis and invasiveness [33,34,35,36]. The inhibition of CD73 activity and the pharmacological blockade of ARs decreased cell adhesion to the extracellular matrix of U138MG glioblastoma cells, demonstrating the pivotal role played by extracellular adenosine in controlling aggressive traits of GBM [37,38]. Furthermore, the activation of selective AR subtypes has been related to the promotion of the epithelial–mesenchymal transition (EMT) in GBM and other types of cancer [34,39,40]. The EMT is a complex process in which cell phenotype switches from the epithelial to mesenchymal one. Since the origin of glioma tumor cells is different from that of epithelial tumors, it has recently been proposed to use the new concept of glial–mesenchymal transition (GMT) [41,42]. The deregulation of this process has been associated with an increase in cancer aggressive traits [39]. 

Even though ADO producing enzyme and the role of AR in GBM biology have been broadly investigated, the effects of high levels of ADO on GBM aggressiveness and cells cross-talk with other cell types of TME have not yet been elucidated. Therefore, in the present study, a glioblastoma cell line (U343MG) was used as a model to test the direct effects of extracellular ADO high concentration in the promotion of GBM aggressive traits. Furthermore, the direct and indirect effects of the nucleoside on the crosstalk between tumor cells and MSCs were investigated.

## 2. Results

### 2.1. Glioblastoma Cells

#### 2.1.1. ADO Increased the Expression of Stemness Genes and the Motility of Tumoral Cells

**Figure 1:**
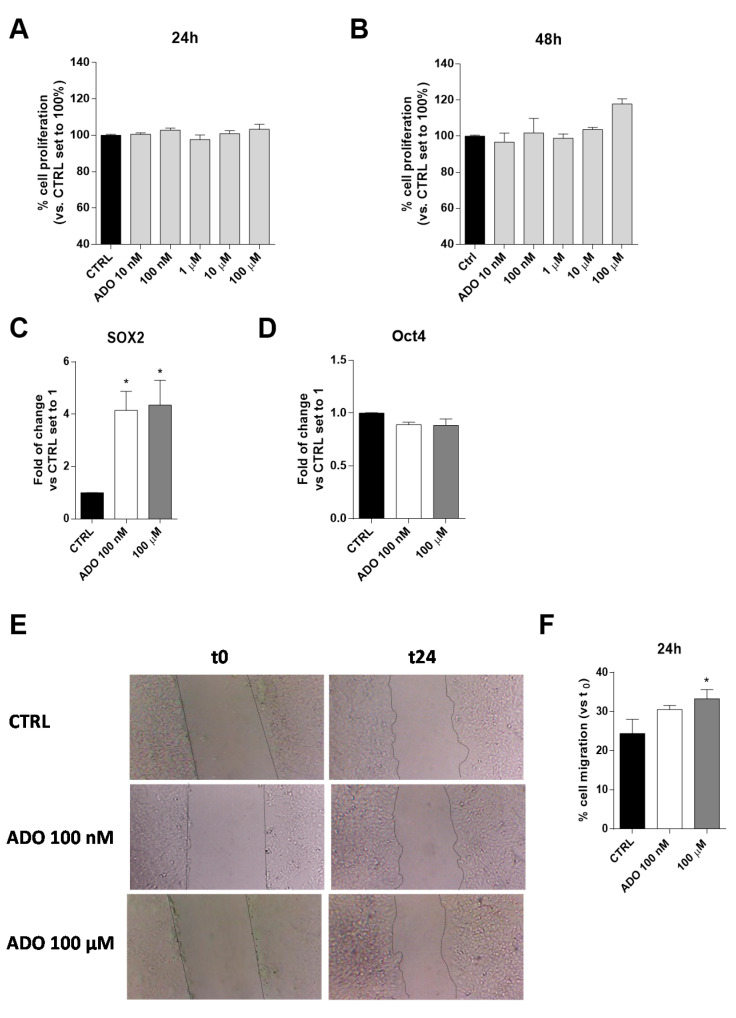
Effects of ADO on U343MG cell proliferation, expression of stemness genes and cell motility. (**A**,**B**) U343MG cells were treated with different concentrations of ADO (10 nM to 100 µM) for: 24 h (**A**); or 48 h (**B**). At the end of the treatment, cell proliferation was evaluated, as described in Section 4.3. The data are expressed as percentages relative to untreated cells (CTRL), which were set at 100% (mean ± SEM; N = 3). (**C**,**D**) The total RNA was extracted from U343MG cells after treatment with ADO (100 nM and 100 µM) for 48 h, and the relative mRNA quantification of the markers SOX2 (**C**) and Oct4 (**D**) was performed by RT-PCR, as described in Section 4.4. The data are expressed as fold changes with respect to basal value set to 1 (mean values ± SEM, N = 2). (**E**,**F**) U343MG cells were treated with ADO (100 nM or 100 µM) and the healing of the wound was evaluated in the scratch assay. (**E**) Representative images of the scratch wounds at 0 and 24 h. (**F**) The data are expressed as percentage of gap closure after 24 h of treatment compared to the untreated cells (CTRL), set to 100%. The data are represented as the means ± SEM of at least of three independent experiments. The significance of differences was determined by one-way ANOVA, followed by Bonferroni’s post hoc test: * *p* < 0.05 vs. CTRL.

ADO is an immunosuppressive metabolite produced at high levels within the TME and contributes, through different mechanisms, to the progression of the tumor itself [43,44]. The effects of a wide range of ADO concentrations (from 10 nM to 100 µM) on U343MG proliferation and viability were evaluated after 24 or 48 h of cell treatment. The U343MG human glioblastoma cell line has been widely used to investigate the pathophysiological mechanisms of glioblastoma [45,46]; herein, we selected this cell line as a representative model to investigate the effects of ADO in the tumor microenvironment. The obtained results show extracellular ADO did not significantly increase cell proliferation after 24 or 48 h of treatment, even if a slight increase could be appreciated at the highest ADO concentrations at 48 h (Figure 1A,B). 

To deeply investigate the effects of ADO on GBM biology, we selected two ADO concentrations: a low concentration (100 nM), similar to the ADO physiological concentrations [31], and a maximal concentration (100 μM), able to promote not only metabolic effects but also to guarantee the activation of all the AR subtypes. These concentrations will be maintained in all the following experiments. 

Actually, among several features determining the aggressiveness of gliomas, the expression of specific stemness genes, such as SOX2 and Oct4, correlates with a poor prognosis [47]. For this reason, the effects of ADO administration on these gene expression were evaluated. ADO significantly increased the gene expression of SOX2 (*p* < 0.005), without affecting the Oct4 expression (Figure 1C,D). 

Another pivotal feature of glioblastoma aggressiveness is its high motility that has been related to its metastatic potential [48]. Thus, ADO effects on cell migration were evaluated, through Scratch assay (Figure 1E,F). Challenging cells with ADO for 24 h caused an increase of U343MG motility, as also observed by optical microscopy (Figure 1E). The effects on cell motility were dependent on ADO concentration, with the highest concentration (100 µM) leading to a significant increase of gap-closure (Figure 1F).

#### 2.1.2. ADO Promoted a Partial Activation of GMT

**Figure 2:**
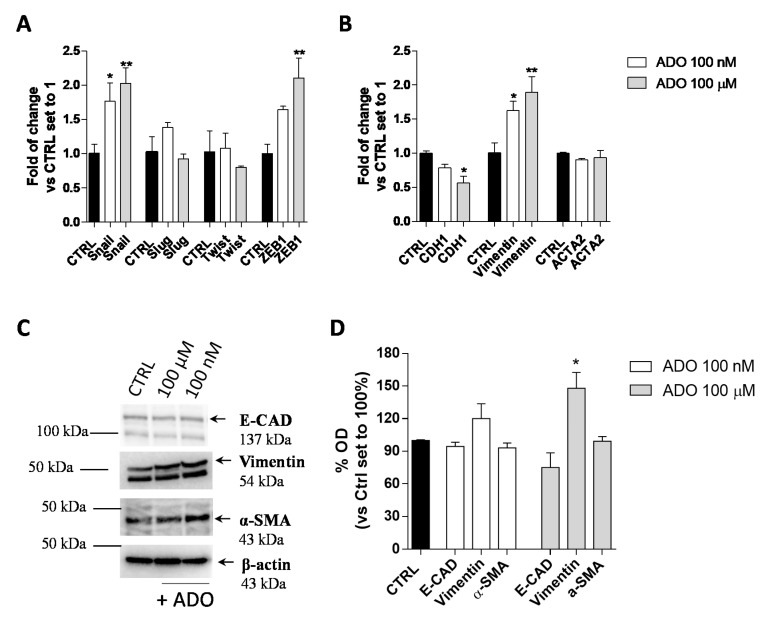
ADO modulation of GMT process in glioma cells. U343MG cells were treated with ADO (100 nM or 100 µM) for 72 h. (**A**,**B**) The mRNA expression levels of GMT master genes (Slug, Snail, Twist and ZEB1) (**A**) and the epithelial (CDH1) and mesenchymal (Vimentin and ACTA2) markers (**B**) were determined by Real-Time RT-PCR. The data are expressed as fold changes with respect to basal value set to 1 and are the mean values ± SEM of two independent experiments. (**C**,**D**) U343MG cells were treated as described above and the protein expression of Epithelial (E-CAD) and Mesenchymal markers (Vimentin and α-SMA) were evaluated by Western blotting. (**C**) One representative blot for each protein is presented and (**D**) the bar graph shows the densitometric analysis of the Western blot performed using ChemiDocTM XRS+ System (BioRad, Hercules, CA, USA). The data are expressed as the fold change vs. the CTRL levels, which were set to 1 and are the mean values ± SEM of three different experiments. The significance was determined by one-way ANOVA, followed by Bonferroni’s post hoc test: * *p* < 0.05, ** *p* < 0.01 vs. CTRL.

The EMT plays an important role in promoting cancer aggressive traits, such as invasiveness and the ability to develop metastases. In the transition, a shift in the expression of epithelial genes to a mesenchymal gene repertoire occurs [49]. Accordingly, the effects of extracellular ADO on the induction of GMT in glioblastoma cells were explored. First, the gene expression of transcription factors such as Snail (SNAI1), Slug (SNAI2), Twist and ZEB1, which are considered the master gene regulators of the GMT process, in response to ADO treatment was evaluated (Figure 2A). The treatment of U343MG cells with 100 nM ADO slightly affected the expression of EMT transcription factors producing only a significant increase of Snail expression (1.8 ± 0.3-fold change; *p* < 0.05). When ADO was used at 100 µM concentration, a significantly increase of Snail (2.0 ± 0.2-fold change; *p* < 0.01) and ZEB1 (2.1 ± 0.3-fold change; *p* < 0.01) expression was observed, without effects on the Slug and Twist gene expression. 

Then, the gene and protein expression of the CDH1, as an epithelial marker, and Vimentin and α-smooth muscle actin (α-SMA; coded by ACTA2 gene), as mesenchymal markers, were quantified (Figure 2B–D) by RT-PCR (Figure 2B) and Western blot analysis (Figure 2C,D). At low concentration, ADO (100 nM) was not able to promote a complete induction of the GMT process. Conversely, the treatment with a higher ADO concentration (100 µM) produced a significant increase in the Vimentin gene expression (1.9 ± 0.2-fold change; *p* < 0.05) and a decrease in the CDH1 gene expression (0.6 ± 0.1-fold change; *p* < 0.05 Figure 2B). These results were in accordance with the ability of a high ADO concentration to significantly increase the Vimentin protein expression (148.0 ± 14.55%, *p* < 0.05), even if it was not sufficient to significantly affect the CDH1 and α-SMA protein levels (Figure 2C,D).

#### 2.1.3. ADO Modulated the Induction of U343MG GMT through ERK Phosphorylation

**Figure 3:**
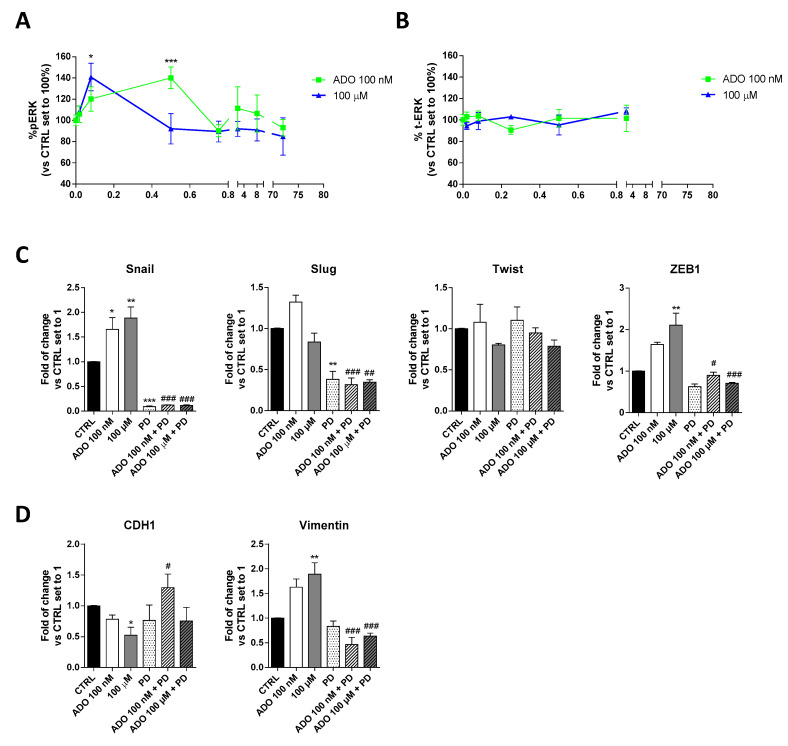
Involvement of ERK 1/2 phosphorylation in ADO-mediated induction of GMT traits. (**A**) Time-course analysis of ERK 1/2 phosphorylation in U343MG cells. U343MG cells were treated with ADO (100 nM and 100 µM) for different time (2 min–72 h), and ERK 1/2 phosphorylation was measured by immuno-enzymatic assay. The data are expressed as the percentage versus untreated cells (CTRL) set to 100% ± SEM of at least three independent experiments performed in duplicate. (**B**) Time-course analysis of total ERK 1/2 in U343MG cells. (**C**,**D**) U343MG cells were treated with ADO (100 nM and 100 µM) in the presence or absence of 1 µM PD184352 for 72 h. mRNA expression levels of GMT master genes (Slug, Snail, Twist and ZEB1) (**C**) and of the Epithelial (CDH1) and Mesenchymal (Vimentin) markers (**D**) were determined by RT-PCR. The data are expressed as fold changes with respect to basal value set to 1 and are the mean values ± SEM of two independent experiments. The significance of the differences was determined by one-way ANOVA, followed by Bonferroni’s post hoc test or two-way ANOVA with Bonferroni correction and two-sided tests for multiple comparisons. * *p* < 0.05, ** *p* < 0.01, *** *p* < 0.001 vs. CTRL; # *p* < 0.05, ## *p* < 0.01, ### *p* < 0.001 vs. corresponding ADO treatment.

Several studies have shown that ERK favors the expression and function of various proteins related to the EMT process, thus promoting tumor progression [50]. To deeply investigate the mechanism involved in ADO induction of GMT process in glioblastoma cells, the time-course of ERK phosphorylation was evaluated (Figure 3A). Specifically, a cell-based immuno-enzymatic assay was performed as previously described [51,52]. ADO, at both concentrations, was able to significantly increase the ERK1/2 phosphorylation without affecting the total-ERK levels (Figure 3B). Despite ADO produced a transient activation of these kinases, the kinetics of phosphorylation slightly differ based on the ADO concentration used. Challenging cells with 100 nM ADO produced a rapid increase of pERK1/2 levels that returned to the basal levels after 5 min; in contrast, the decrease of phosphorylation was slower when the 100 µM ADO concentration was used. These discrepancies could be probably ascribed to a diverse activation of AR subtypes in dependence on ADO concentration and could explain the diverse effects on GMT modulation. 

Then, to verify the involvement of ERK pathways in ADO-mediated GMT-induction, cells were treated with a selective MEK inhibitor (PD-184352, 1 µM), and the gene expression of GMT transcription factors (Figure 3C) and GMT markers (Figure 3D) were analyzed by RT-PCR. As expected, the treatment with PD for 72h caused a significant decrease of Snail (*p* < 0.001) and Slug (*p* < 0.01), without affecting the expression of Twist, ZEB1 and the transition markers, CDH1 and Vimentin. When the MEK inhibitor was used, the changes induced by ADO stimulation were almost completely counteracted. In particular, the Snail (*p* < 0.001 vs. ADO 100 nM; *p* < 0.05 vs. ADO 100 µM) and ZEB1 (*p* < 0.001 vs. ADO 100 nM; *p* < 0.001 vs. ADO 100 µM) expression were significantly reduced in the presence of both ADO concentration (Figure 3C). The inhibition of ERK phosphorylation prevented also the ADO increase of Vimentin expression (1.63 ± 0.17-fold vs. 1.89 ± 0.23-fold, *p* <0.001, ADO 100 nM; 0.46 ± 0.14-fold vs. 0.63 ± 0.06-fold, *p* <0.001, ADO 100 µM) and the decrease of CDH1 gene expression (0.78 ± 0.06-fold vs. 1.30 ± 0.22-fold, *p* <0.05, ADO 100 nM; 0.52 ± 0.13-fold vs. 0.7540 ± 0.22-fold ADO 100 µM; Figure 3D), confirming that ERK signaling is indeed required for GMT induction driven by ADO. 

### 2.2. Mesenchymal Stromal Cells: Effects of ADO on Cell Proliferation and Cytokine Release

**Figure 4:**
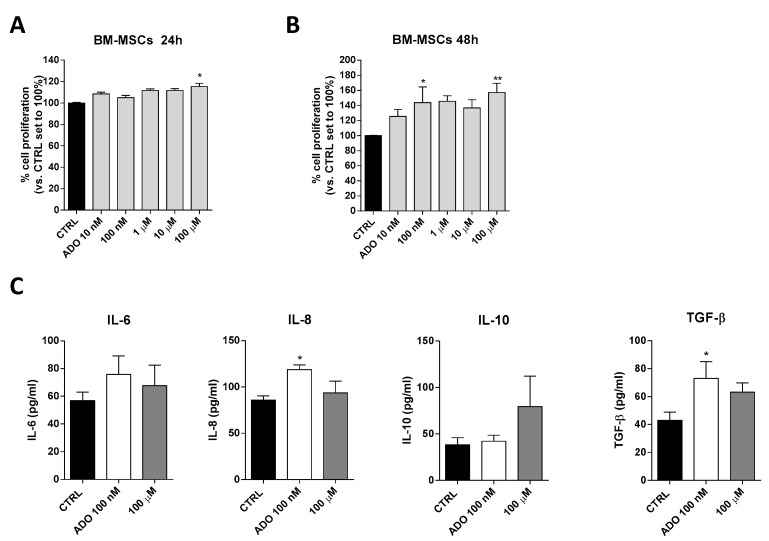
ADO effects on BM-MSCs proliferation and BM-MSCs cytokines release. (**A**,**B**) BM-MSCs were treated with different concentrations of ADO (10 nM to 100 µM) for 24 h (**A**) or 48 h (**B**) in complete medium. At the end of the treatment period, cell proliferation was evaluated as described in Section 4.3. The data are expressed as percentage relative to untreated cells (CTRL), which were set at 100% (mean ± SEM; N = 3). (**C**) BM-MSCs were treated in serum-free medium with ADO (100 nM and 100 µM) for 48 h. At the end of treatment, the IL-6, IL-8, IL-10 and TGF-β levels in the medium were quantified using commercial ELISA kits. The data are reported as the mean values ± SEM of three independent experiments. The significance of the differences was determined by one-way ANOVA, followed by Bonferroni’s post-hoc test: * *p* < 0.05, ** *p* < 0.01 vs. the CTRL.

Based on ADO ability to affect glioblastoma aggressiveness, we investigated the effects of ADO on cell-to cell communication in glioblastoma microenvironment focusing in particular on MSCs that play a pivotal role in the control of immune system in TME. First, the effects of a wide range of ADO concentrations (from 10 nM to 100 µM) on BM-MSCs proliferation were evaluated after 24 (Figure 4A) and 48 h (Figure 4B) of cell treatment. ADO exerted biphasic effects on BM-MSC proliferation promoting a significant peak of proliferation at 100 nM (*p* < 0.05) and 100 µM (*p* < 0.01). Notably, the ADO effects on proliferation of BM-MSCs was greater than that observed on GBM cells (Figure 1B). 

Within the TME, the interactions between MSCs and tumor cells involve several MSC-secreted signaling molecules, such as cytokines and growth factors that stimulate signaling pathways involved in tumor cell survival, growth, motility, and immune escape [53]. Among these, a pivotal role is played by IL-6 and TGF-β as inducers of GMT and cancer growth, IL-10 as a modulator of immune response and the chemoattractant IL-8 as a promoter of GBM motility [53]. The ability of BM-MSCs to release these cytokines in response to extracellular stimuli has been reported previously [54]. To investigate the effects of ADO on BM-MSC secretome composition, the BM-MSCs were treated with two ADO concentrations and the levels of secreted cytokines were analyzed (Figure 4C). Challenging BM-MSCs with 100 nM ADO produced a significant alteration of IL-8 (*p* < 0.05) and TGF-β (*p* < 0.05) levels (Figure 4C). Conversely, the treatment with ADO 100 µM did not produce a significant modification of IL-6, IL-10, IL-8 and TGF-β cytokine secretion (Figure 4C).

### 2.3. GBM and MSC Cross-Talk

#### 2.3.1. ADO-Treated MSC Secretome Increased Tumoral Cell Proliferation

**Figure 5:**
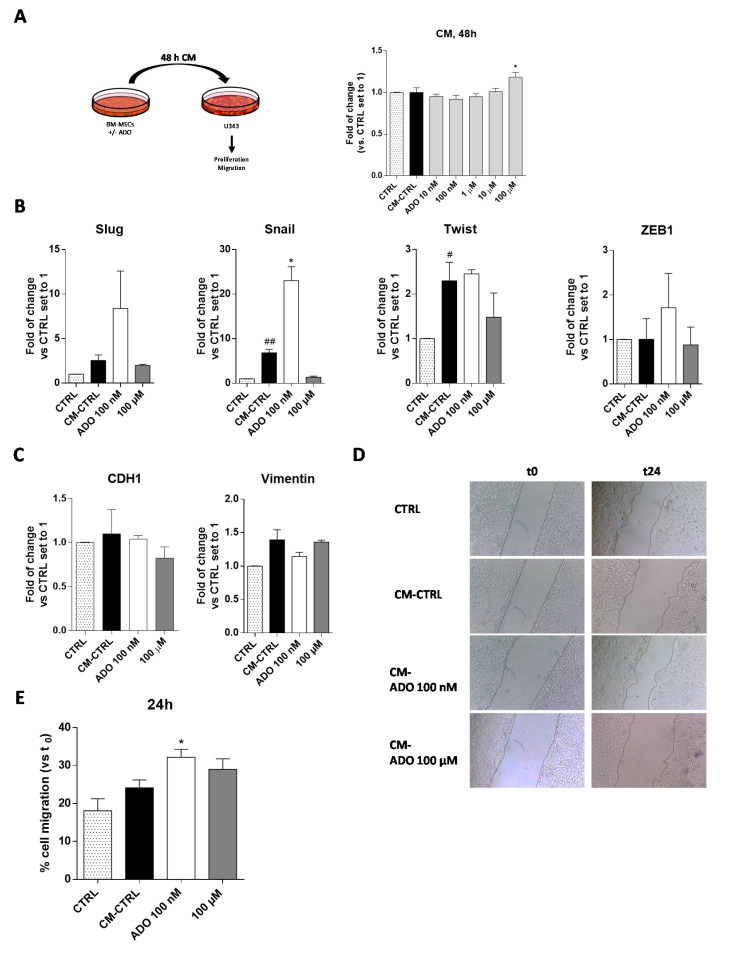
ADO modified the BM-MSC secretome affecting the U343MG proliferation, GMT traits and cell motility. (**A**) U343MG cells were grown in 80% U343MG culture medium + 20% BM-MSC medium (CTRL), CM obtained from untreated cells (CM-CTRL) or cells treated with ADO for 48 h. At the end of the treatments, cell proliferation was evaluated using the MTS assay. The data are expressed as the percentage versus the CTRL, which was set to 100%, and they are presented as the mean values ± SEM of three independent experiments, each performed in duplicate. (**B**,**C**) U343MG cells were treated as described above and, after 72 h of treatment, mRNA expression levels of Slug, Snail, Twist, ZEB1, CDH1 and Vimentin were determined by RT-PCR. The data are expressed as fold changes with respect to untreated cells set to 1 and are the mean values ± SEM of two independent experiments. (**D**,**E**) U343MG cells were treated as above, and representative images (**D**) of the scratch wounds at 0 and 24 h after treatment are reported. (**E**) The data are expressed as percentage of gap closure after 24 h of treatment compared to the CTRL set to 100%. The data are represented as the means ± SEM of at least two independent experiments performed in triplicate. The significance of the differences was determined by one-way ANOVA, followed by Bonferroni’s post-hoc test: # *p* < 0.05, ## *p* < 0.01 vs. CTRL; * *p* < 0.05 vs. CM-CTRL.

To deeply investigate the role played by the extracellular ADO in the cross-talk of MSC and GBM cells, the simplest model consists in the use of conditioned medium (CM) released by MSCs to assess its influence on GBM cell aggressive traits [55,56]. Accordingly, the BM-MSC cells were treated for 48 h with a wide range of ADO concentrations in serum-free medium; at the end, the CM was collected and applied to U343MG to evaluate the effect of the released factors on tumor cell growth (Figure 5A). The CM derived by the BM-MSCs without treatment (CM-CTRL) did not significantly affect GBM cell proliferation. Interestingly, the addition of CM derived by BM-MSCs treated with ADO at high concentration (100 µM) significantly enhanced the U343MG proliferation (*p* < 0.05; Figure 5A). Of note, after 48 of BM-MSC treatment, the amount of exogenous ADO in the CM could be drastically reduced. Thus, the main effects of the CM could be probably ascribed to a modification of soluble factor content.

#### 2.3.2. ADO-Treated MSC Secretome Increased GMT Traits

The CM of MSCs derived from adipose tissue induces the transition to a more invasive GBM cell phenotype modulating the EMT [57]. However, it is well known that the secretome of MSCs derived from diverse sources differently affected the GBM properties [53]. Herein, we first evaluated the effects of BM-MSCs derived CM on the regulation of GMT transcription factors (Figure 5B) and GMT markers (Figure 5C) in U343MG. BM-MSC derived CM promoted a significant increase in Snail and Twist gene expression (*p* < 0.001 and *p* < 0.05, respectively), without affecting the expression of Slug and ZEB1. These effects did not result in a significant modification of vimentin and CDH1 gene expression. The lack of effects on CDH1 gene expression in response to the increase of Snail gene might be related to the activation of other pathways that could negatively regulate the Snail activity such as GSK-3β or PDK1, which are able to promote Snail protein phosphorylation facilitating its proteasomal degradation [58,59,60]. Of note, the modification of cAMP levels and CREB activation can positively regulate the CDH1 expression [61,62,63], counteracting the effects of Snail. As reported above, the treatment of BM-MSCs with ADO produced a modification of MSC secretome. Challenging cells with CM derived by the BM-MSCs treatment with ADO 100 nM caused a significant increase of Snail gene expression (*p* < 0.05), in accordance with the increase of TGF-β levels in BM-MSCs secretome (Figure 4B). However, the promotion of Snail gene expression was not sufficient to promote a complete GMT transition as evidenced by the failure of CDH1 and vimentin gene expression modification (Figure 5C). 

#### 2.3.3. ADO-Treated MSC Secretome Increased Motility of Tumoral Cells

One of the main features supporting GBM aggressiveness is its high invasiveness [48]. The GMT process, as well as soluble factor release in the TME, could directly or indirectly influence GBM motility [64]. Herein, a scratch assay was performed to assess the U343MG cell motility mediated by the BM-MSC CM alone or after the treatment with ADO (Figure 5D,E). The results show that the CM-CTRL did not enhance the U343MG motility; however, the treatment of BM-MSCs with ADO (100 nM) significantly increased the motility of glioblastoma cells (*p* < 0.05, Figure 5E). This effect was in accordance with the increase of IL-8 release, which has chemoattractant properties [65], in the BM-MSC secretome (Figure 4B). Conversely, the CM derived by the BM-MSC treatment with ADO 100 μM did not produce significant effects on U343MG cell motility (Figure 5E).

#### 2.3.4. ADO Modified the MSC-GBM Cross-Talk Promoting the GBM Proliferation and Invasiveness

**Figure 6:**
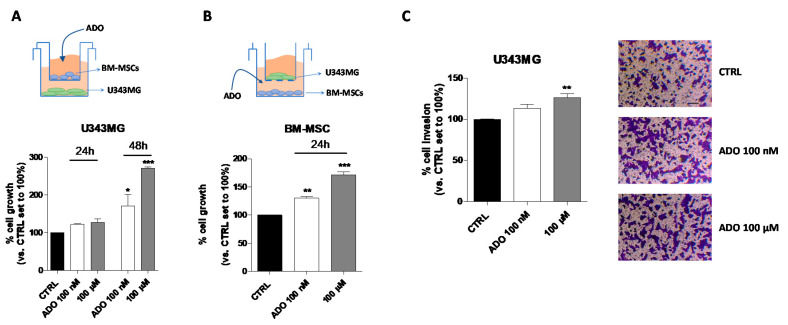
Effects of ADO in the BM-MSC and GBM cell cross-talk. (**A**) The image depicts the contact-independent transwell cultures with BM-MSCs in the upper chamber and U343MG in the lower chamber. U343MG cell proliferation was evaluated after 24 and 48 h with Crystal violet, as reported in Section 4.9. (**B**) The image depicts the contact-independent transwell cultures with U343MG in the upper chamber and BM-MSCs in the lower chamber. BM-MSCs cell proliferation was evaluated after 48 h with Crystal violet, as reported in Section 4.9. (**C**) U343MG cells were seeded in the upper chamber and BM-MSCs treated with ADO (100 nM and 100 µM) in the lower chamber. After 24 h, U343MG cell invasion was analyzed using Matrigel basement membrane transwell system, as described in Section 4.10. Representative images are shown. Cell migration was quantified by counting the number of cells that migrated into the lower surface of the transwell membrane. The data are reported as percentage of cell invasion versus the CTRL set to 100% and are the means ± SEM of three independent experiments. The significance of the differences was determined by one-way ANOVA, followed by Bonferroni’s post-hoc test. * *p* < 0.05, ** *p* < 0.01, *** *p* < 0.001 vs. CTRL.

Even though the use of CM could represent a model to investigate the MSC-GBM crosstalk, it is not enough to fully elucidate the role of the nucleoside in direct cell–cell communication. Thus, a co-culture system of GBM–MSC cells was set up (Figure 6A–C), and the proliferation and invasiveness of GBM cells were assessed in these experimental conditions. First, the influence of ADO in the extracellular microenvironment as a modulator of GBM (Figure 6A) and BM-MSC (Figure 6B) cell growth was analyzed by crystal violet assay. U343MG and BM-MSCs were seeded in two separate compartments and a transwell culture system allowed the exchange of soluble factors between the cells. First, U343MG were seeded in the lower compartment, and BM-MSCs treated with ADO (100 nM or 100 µM) were cultured in the upper compartment (Figure 6A). The U343MG cell proliferation was evaluated after 24 or 48 h of cell treatment. The presence of ADO in the extracellular space caused a significant increase in cell growth only after 48 h of cell treatment. Challenging U343MG cells with BM-MSC CM alone or ADO treatment at different concentration did not significantly enhance the glioma proliferation (Figure 1A,B and Figure 5A); interestingly, the communication of BM-MSCs and U343MG in the presence of ADO caused a significant increase of glioma cell growth, highlighting the importance of soluble factors released in the TME. 

Then, the effects of the nucleoside on BM-MSCs proliferation in a similar co-culture system were evaluated (Figure 6B). ADO (100 nM or 100 µM) was administrated to the BM-MSCs in the lower compartment and U343MG were seeded in the upper one. The 24-h treatment was sufficient to produce a significant increase of BM-MSC proliferation at both ADO concentrations used (*p* < 0.01 and *p* < 0.001, respectively; Figure 6B). These effects were similar to those obtained in the absence of U343MG (Figure 4A), demonstrating that the main effects on BM-MSC growth are mediated by the extracellular ADO itself rather than to other factors released by glioma cells.

Finally, the effects of the nucleoside in the modulation of BM-MSC-U343MG cross-talk were evaluated analyzing the glioma cell invasiveness properties (Figure 6C). BM-MSCs were seeded in the lower compartment and treated with ADO (100 nM and 100 µM), while U343MG were seeded in the transwell upper compartment on a Matrigel coating. Although ADO increased the invasive capacity of U343MG cells at both concentrations, the effects became significant only when a 100 µM concentration was used (*p* < 0.01; Figure 6C). Interestingly, a low concentration of ADO (100 nM) was sufficient to modify the BM-MSC cell secretome increasing the U343MG cell motility (Figure 5E). However, in the co-culture system, a higher ADO concentration is required, probably due to the simultaneous ADO action on U343MG and BM-MSCs cells, leading to the need for a higher concentration to obtain similar effects.

## 3. Discussion

Glioblastoma is a malignant brain tumor displaying high recurrence rates because of its aggressiveness and resistance to chemotherapy and radiotherapy. Although the mechanisms governing the glioma pathogenesis and development have been extensively studied, recently the role of TME has emerged as a pivotal player in modulating GBM aggressiveness and invasiveness [66]. The TME affects glioma cells by the interactions with other types of cells such as MSCs [53,67] or soluble factors released by cancer cells as well as other types of cells in the TME. In this context, a crucial role has been proposed for ADO and the entire purinome [30]. Studies have suggested that ADO plays a significant role in cancer development and progression by attenuating the immune response [68]. However, the effects exerted by high concentrations of ADO in the TME on glioma biology are still unclear. This study aimed to provide new insight into the effects of prolonged administration of extracellular ADO on isolated human glioblastoma cells and BM-MSCs as well as on their cross-talk. Our results demonstrate that ADO can promote the insurgence of more aggressive traits on glioma cells in vitro. Notably, its administration in the extracellular space modifies the release of soluble factors by BM-MSC prompting also indirectly the glioma aggressive traits.

Evidence suggests that ADO derived by different sources can have distinct functions in cancer cells through either canonical adenosine receptor-mediated pathways (A_1_, A_2A_, A_2B_ and A_3_ AR), or receptor-independent intrinsic mechanisms such as the activation of AMP-dependent protein kinase (AMPK) [69,70], based on ADO concentration. Interestingly, most of the studies on ADO effects in cancer cells use a single administration with concentrations up to 10–20 mM [69,71,72,73]. Notably, ADO is rapidly metabolized, and, even though the extracellular levels significantly increase under several stressful conditions, they only reach micromolar concentrations in the glioma TME [74,75]. Herein, to better reproduce the pathological role of the chronic exposure of glioma cells to micromolar concentrations of ADO in TME, a recurrent administration of the nucleoside was performed. 

Studies on the nucleoside influence on different tumor cell lines and on the other cells of the TME are still conflicting. Some literature data indicate that ADO plays a prominent role in multiple areas of glioma pathogenesis, including promoting cell growth, angiogenesis and invasiveness [33,34,35]. Our results demonstrate that the chronic exposure of up to micromolar extracellular ADO concentration does not significantly modify glioblastoma cell proliferation. The reduction of ADO production by the CD73 inhibition causes the decrease of glioma proliferation in vitro and in vivo [33,76]. Similarly, a single administration of 100 µM ADO concentration enhances the human U138MG glioma cell proliferation in vitro [77]. However, previous studies have demonstrated that millimolar levels of extracellular ADO significantly suppress the growth of tumors in the pancreas, liver and colon [71,72,73], demonstrating the debated role of ADO on cancer cell biology based on its concentrations, time of treatment, AR expression levels and cancer cell type [78].

Besides the high proliferation rate, one feature that characterizes glioma aggressiveness is the expression of different stem cell markers such as SOX2 and Oct4 [79]. ADO treatment significantly enhanced the expression of SOX2 without affecting the expression of Oct4. SOX2 is a well-established master gene involved in the maintenance of tumor stem cell such as phenotype, and its silencing has been associated to a reduction of glioma tumorigenicity [80]. Thus, the increase of SOX2 levels demonstrates the ability of ADO to partially change the GBM hallmarks promoting the expression of a gene associated with cancer aggressiveness. 

One of the main features of cancer metastatic cells is the acquisition of higher motility [50]. ADO treatment has been reported to promote the migration of breast cancer cells [81]. Moreover, the activation of A3 AR has been reported to increase the motility of glioma stem cells [34]. Herein, we demonstrate that the prolonged exposure to a high ADO concentration (100 µM) promotes the glioma motility in vitro. This result is in accordance with the ability of CD73 to enhance cell adhesion through ADO production, thereby promoting cell migration and invasion in different cancer cells and in glioma cells [29,38,76,82]. 

ADO signals as well as the activation of its receptor can modulate the EMT in different types of cancer such as head, gastric and lung cancer, resulting in a more invasive phenotype [39,83,84]. Accordingly, high ADO concentrations are able to promote the gene expression of Snail and ZEB1, two master gene regulators of GMT induction. The variation of gene expression not completely reflect the profile of GMT protein expression demonstrating that, even though ADO could induce some traits of the mesenchymal phenotype, it is not able to trigger a complete transition. These results are in accordance with the ability of ADO to promote the Vimentin gene expression in C6 rat glioma cells [76] and strengthen the pivotal role exerted by extracellular ADO in the control of glioma aggressive traits in human cells. Interestingly, ADO regulates the induction of GMT in U343MG almost in part through the increase of ERK1/2 phosphorylation. The EMT process is tightly controlled, and several intracellular mechanisms are involved in its regulation including ERK1/2 phosphorylation [54]. The ability of ADO to increase ERK 1/2 phosphorylation in airway epithelia cells has been reported previously [85]. Furthermore, the activation of ADO receptors such as A_2B_AR has been linked to the increase of ERK phosphorylation and EMT induction [39], supporting the central role of MAPK pathway in ADO-mediated GMT induction in glioma cells. 

The TME is heterogeneous and is also composed of other cell types such as fibroblasts, endothelial cells, GAMs, TAFs and MSCs that can be affected by the presence of extracellular ADO. Accumulating evidence suggests that MSCs can produce ADO [86,87], playing a crucial role in the regulation of immune system [44]. Our data show that ADO promotes BM-MSC proliferation, but the trend does not demonstrate a concentration-dependent effect, probably due to the activation of different receptor subtypes that mediate different and opposite functions. Interestingly, the presence of ADO does not extensively modify the cytokine secretion from BM-MSCs. However, the nucleoside increases the release of TGF-β, which is a growth factor that may stimulate various signaling pathways related to tumor cell growth and motility [14]. Accordingly, the ability of ADO to promote the release of TGF-β has been reported in other types of cells [88].

There is increasing evidence of the presence of MSCs in GBM recruited from local sites or from BM [89]. The MSC role in glioma progression is still debated considering that both anti-tumor and pro-tumor effects have been reported based on the source of MSCs, the cellular model used and the type of MSC-GBM interaction [53]. However, the puzzle is complicated by the presence of soluble factors in TME that could affect each single cell type but also the cross-talk between different cells in TME. This evidence prompted us to investigate the role of ADO in glioma-MSCs cross-talk. For this purpose, initially, the CM derived from ADO treated BM-MSC was used to assess if the BM-MSC nucleoside treatment can affect the U343MG glioma proliferation and mobility. The ADO treated BM-MSC CM promotes cell proliferation when used at high concentrations. Interestingly, a low ADO concentration (100 nM) treatment is sufficient to modify the CM and significantly increased the motility of glioblastoma cells. These effects are in accordance with the ability of ADO to modulate the release of IL-8 from BM-MSCs, which also modulates cell motility in other types of cancer [90,91]. 

It is well known that MSCs directly or indirectly promote the EMT process in different types of cancer [92]. Recently, Iser et al. [57] demonstrated that the CM derived by adipose-derived MSCs promote the EMT process in C6 cells. These effects are supported by the ability of MSCs to secrete soluble factors such as IL-6 and TGF-β [54], which are two key players in the promotion of a transition to a mesenchymal phenotype [93]. Our results support the capacity of BM-MSC to release soluble factors able to promote the expression of the transcription factors such as Snail and Twist. These genes can in turn tune the induction of a mesenchymal phenotype. Interestingly, ADO modifies the BM-MSC secretome supporting the induction of a more aggressive phenotype in U343MG cells through the increase of both the GMT master gene transcription and the cell motility. However, the presence of ADO is not enough to support a complete transition supporting the idea that ADO could be probably only one of the many soluble factors in the TME able to modify the GBM–MSC cross-talk.

The communication in a cell system is bidirectional, and the effects evoked by the MSC-CM could not fully reflect the interplay between the MSCs and glioma cells [53]. Accordingly, different effects of the direct or soluble factor-mediated interaction of GBM stem cells with the human umbilical cord-derived MSCs have been reported [56]. Thus, to deeply investigate the ADO effects on GBM–MSC communication, we set-up a co-culture system. The results demonstrate that increasing concentrations of ADO, in the TME, support GBM cell proliferation and invasiveness directly and indirectly through the modification of GBM–MSC cross-talk. To note, a higher ADO concentration is required in the co-culture system to obtain similar effects shown on glioma cells, probably because ADO is distributed between the two cell types (U343MG and BM-MSCs cells). The use of a simple cell model is crucial to better clarify the effects of a single molecule; however, our results further highlight the importance to use an advanced cellular model to better explain the role of soluble factors in TME.

## 4. Materials and Methods

### 4.1. Materials

Human bone marrow MSCs (BM-MSCs) and glioblastoma stem cells (U343MG) were purchased by CLS (CLS Cell Lines Service GmbH, Eppelheim, Germany). Cell culture medium and Adenosine were purchased from Sigma Aldrich (Milan, Italy). RNeasy^®^ Mini Kit was purchased from Qiagen S.p.A., Milan, Italy. The iScript cDNA synthesis kit was furnished by Bio-rad s.r.l., Milan, Italy Fluocycle^®^ II SYBR^®^ was obtained from Euroclone s.p.a. (Milan, Italy). PD-184352 (2-(2-Chloro-4-iodophenylamino)-N-cyclopropylmethoxy-3,4-difluorobenzamide, Sigma-Aldrich, Milan, Italy) was used as inhibitor of Extracellular Regulated Kinase (ERK).

### 4.2. Cell Cultures

BM-MSCs were cultured in the appropriate Mesenchymal Stem Cells Expansion medium (Sigma-Aldrich, Milan, Italy) and incubated at 37 °C in 5% CO_2_ and 95% air. The medium was changed to remove non-adherent cells every 3–4 days and the cells were used at Passages 1–6. The human glioblastoma cell line, U343MG, was maintained in Eagle’s Minimum Essential Medium (EMEM) with 2 mM L-glutamine adjusted with 1.5 g/L sodium bicarbonate and supplemented with 10% FBS, 100 U/mL penicillin, 100 μg/mL streptomycin, 1% non-essential amino acids and 1 mM sodium pyruvate at 37 °C in 5% CO_2_. The medium was changed to remove non-adherent cells every 3–4 days.

### 4.3. Cell Viability Assay (MTS)

U343MG and BM-MSCs were seeded in 96-well microplates (5000 and 3000 cells/well, respectively) and treated with different concentrations of ADO (10 nM to 100 µM). ADO treatment was repeated every day. Following the treatment period, cell proliferation was determined using an MTS assay (CellTiter 96 AQueous One Solution Cell Proliferation Assay kit; Promega, Milan, Italy) according to the manufacturer’s instructions. The absorbance of formazan at 490 nm was measured in a colorimetric assay with the EnSightTM multimode plate reader (Perkin Elmer, Waltham, MA, USA). U343MG cells were seeded in 96-well microplates at a density of 3000 cells/well and treated with Conditioned Medium (CM), collected from the BM-MSCs cells previously treated with ADO (100 nM or 100 µM), for 48 h. The CM was centrifuged at 500× *g* and used at 20% in complete medium. Following the treatment period, cell proliferation was determined using an MTS assay, as previously reported.

### 4.4. Glioblastoma and MSCs Real-Time RT-PCR Analysis

U343MG cells were incubated with ADO (100 nM or 100 µM), in the presence or absence of 1 µM PD-184352, for 72 h or with CM at 20% of the total complete medium for 72 h. At the end of treatments, cells were collected, and total RNA was extracted using the Rneasy Mini Kit (Qiagen, Hilden, Germany). The RNA purity was checked measuring the A260/280 ratio. cDNA synthesis was performed with 500 ng of RNA using i-Script cDNA synthesis kit (Bio-Rad, Minal, Italy). RT-PCR reactions consisted of 25 µL Fluocycle^®^ II SYBR^®^ (Euroclone, Milan, Italy), 1 µL of both 10 µM forward and reverse primers, 3 µL of cDNA and 20 µL of H2O. The primer sequences, product size and annealing temperature are listed in Table 1 [94]. HPRT1 primers was purchased by Biorad (qHsaCIP0030549). The mRNA levels for each sample were normalized against β-actin or HPRT1 mRNA levels, and the relative expression was calculated by using the methods of the ΔΔCt value.

### 4.5. Cell Migration Assay

U343MG cells were seeded in 96-well plates and grown to 90% confluence. Then, a scratch was made through the cell layer using a sterile micropipette tip. After washing with PBS, cells were treated with ADO (100 nM or 100 µM) in medium with 1% FBS or with CM collected as described above. The images of the wounded area were captured immediately after the scratch (t0) and 24 h later (t24) to monitor cell migration into the wounded area. Photographs were then taken at 4× magnification on an inverted microscope. The wound healing abilities were quantified by measuring the percentage of gap closed. The data were analyzed with Image J software.

### 4.6. Western Blot Analysis of Protein Expression

U343MG cells (500.000 cell/cm^2^) were treated with ADO (100 nM or 100 µM) for 72 h. The treatment was refreshed every 24 h. At the end of treatment, cells were harvested and lysed for 2 h at 4 °C by the addition of 200 µL of RIPA buffer. Equal amounts of the cell extracts (40 μg of proteins) were diluted in Laemmli solution, resolved by SDS-PAGE (7.5%), then transferred to PVDF membranes and probed overnight at 4 °C with primary anti-actin smooth antibody (diluted 1:400; Monoclonal Anti-Actin, α-Smooth Muscle, A2547, Sigma-Aldrich), anti-E-cadherin antibody (diluted 1:200, sc-7870, Santa Cruz Biotechnology, Dallas, TX, USA) and anti-vimentin antibody (diluted 1:2000, #5741; Cell Signaling Technology, Danvers, MA, USA). The primary antibody was detected using an appropriate secondary antibody. The peroxidase was detected using a chemiluminescent substrate (ECL, Perkin Elmer, Waltham, MA, USA), and the images were acquired by ChemiDoc. Immunoreactive bands were quantified performing a densitometric analysis with Image J Software (version 1.41; Bethesda, Rockville, MD, USA).

### 4.7. MAPK (Mitogen-Activated Phosphorylation Kinase) Assays

U343MG cells were seeded at a density of 3000 cells/well in a 96-well plate and treated in complete medium with ADO (100 nM and 100 µM) for different times (1 min to 72 h). Levels of total and phosphorylated extracellular signal-regulated kinases (ERK1/2) were determined by ELISA assays, as previously reported [37]. Briefly, after three washes with wash buffer (0.1% Triton X-100 in PBS), 100 µL of quenching buffer (1% H_2_O_2_; 0.1% sodium azide in wash buffer) were added and incubated for 20 min. The cells were washed with PBS twice, and then 100 µL of blocking solution (1% BSA; 0.1% Triton X-100 in PBS) were added for 60 min. After blocking, cells were washed three times with wash buffer and the specific primary antibodies (anti-phospho ERK1/2, 1:400, sc-7383 Santa Cruz Biotechnology; anti-ERK1/2, 1:400, sc-514302 Santa Cruz Biotechnology, Dallas, TX, USA) were added on at 4 °C. After incubation with secondary HRP-conjugated antibodies, the developing solution was added, and a colorimetric quantification of total and phosphorylated ERK1/2 levels was made. Blanks were obtained by treating cells in the absence of the primary antibody. The relative number of cells in each well was obtained using Crystal Violet solution. The results were calculated by subtracting the mean background from the values obtained from each test condition; values were normalized to the number of cells in each well and were expressed as the percentage of untreated cells (basal).

### 4.8. Quantification of IL-6, IL-8, IL-10 and TGF-β Production

BM-MSCs were treated with ADO (100 nM or 100 µM) for 48 h in serum-free medium. The amounts of cytokines presented in the culture medium (IL-6, IL-10, IL-8 and transforming growth factor beta (TGF-β)) were measured using commercial enzyme-linked immunosorbent assay (ELISA) kits (Cat. Nos. SEA079Hu (IL-6), SEA056Hu (IL-10), SEA080Hu (IL-8) and SEA397Hu (TGF-β), Cloud-Clone Corp, Katy, TX, USA) following the manufacturer’s instructions. After collecting the culture medium, cells were fixed with p-formaldehyde (4%) for 15 min. After three washes, the Crystal Violet solution was added for 20 min. Then, cells were washed with PBS four times and 100 µL of SDS 1% were added. The absorbance was read at 595 nm and used to normalize the reading absorbance in the ELISA kit. 

### 4.9. Glioblastoma and MSCs Co-Culture

U343MG were co-cultured with BM-MSCs in 24-well transwell chamber (0.4-µm pore size polyester (PET) membrane from BD Biosciences, San Jose, CA, USA). U343MG cells were seeded at the density of 3000 cells and BM-MSCs were seeded at the density of 1000 cells. Cells were co-cultured for 24 or 48 h. At the end of the treatment period, cells in the lower compartment were fixed with p-formaldehyde and staining with crystal violet. After 15 min of incubation, the excess dye was removed by performing three washes with PBS. One hundred microliters of 1% SDS solution were added to each well and was incubated for 1 h at room temperature under gentle stirring. The wells were read at 595 nm with the EnSightTM multimode plate reader (Perkin Elmer, Waltham, MA, USA).

### 4.10. Cell Invasion Assay

The effect of ADO on cell invasion was evaluated using a 24-well transwell chamber (8-μm pore size polyester (PET) membrane), as previously reported [95]. The surface of the transwell was coated with Matrigel basement membrane matrix (BD Biosciences, San Jose, CA, USA) (0.32 mg/mL) at room temperature, and BM-MSCs were seeded at the density of 3000 cells in the lower compartment. U343MG cells were suspended in serum-free medium (20,000 cells/300 μL) and added to the upper compartment of the 8-μm pore size insert, while BM-MSCs were treated with ADO (100 nM and 100 μM). The cells were allowed to invade through the matrixes at 37 °C for 24 h. The non-migrating cells of the upper chamber were removed with a cotton bud. The number of invading cells was measured by counting the cells on the lower surface of the transwell membrane after fixing with p-formaldehyde and staining with crystal violet. Pictures of randomly picked light microscope fields were taken (five fields for each filter), and cells were counted using ImageJ Software.

### 4.11. Data Analysis

Graph-Pad Prism (Version 5.00), was used for data analysis and graphic presentation. Data are reported as the mean ± SEM of 2–4 different experiments. Statistical analyses were performed using a one-way ANOVA study followed by the Bonferroni test for repeated measurements. Differences were considered statistically significant when *p* < 0.05.

## 5. Conclusions

The results demonstrate that a soluble factor in the TME, ADO, plays a pivotal role in the modification of glioma biology directly and through the modulation of the paracrine cross-talk of GBM cells with other cells of the TME such as the BM-MSCs. The modification of key intracellular signaling pathways in response to ADO chronic exposure may promote GMT traits, highlighting the importance of the extracellular ADO in the control of glioma aggressiveness. At the same time, we shed light on the importance to deeply investigate not only the cross-talk of GBM cells with other cell types of TME but also the role of a soluble factor in the extracellular space to better understand these complex interactions and find new potential therapeutic targets.

## Figures and Tables

**Table 1:** Primers used for RT-PCR.

Gene	Primer Nucleotide Sequences	Product Size (Base Pairs)
Snail	FOR: 5′-AAGATGCACATCCGAAGCCA-3′ REV: 5′-CATTCGGGAGAAGGTCCGAG-3′	237 bp
Slug	FOR: 5′-TGGTTGCTTCAAGGACACAT-3′ REV: 5′-GTTGCAGTGAGGGCAAGAA-3′	66 bp
Twist	FOR: 5′-ACGAGCTGGACTCCAAGATG-3′ REV: 5′-CACGCCCTGTTTCTTTGAAT-3′	290 bp
ZEB1	FOR: 5′-CCCTTGAAAGTGATCCAGCCA-3′ REV: 5′-AGACCCAGAGTGTGAGAAGCG-3′	354 bp
β-actin	FOR: 5′-GCACTCTTCCAGCCTTCCTTCC-3′ REV: 5′-GAGCCGCCGATCCACACG-3′	254 bp
CDH1	FOR: 5′-AGGGGTTAAGCACAACAGCA-3′ REV: 5′-GGGGGCTTCATTCACATCCA-3′	395 bp
Vimentin	FOR: 5′-CTCTTCCAAACTTTTCCTCCC-3′ REV: 5′-AGTTTCGTTGATAACCTGTCC-3′	134 bp
SOX2	FOR: 5′-CATGAAGGAGCACCCGGATT-3′ REV: 5′-ATGTGCGCGTAACTGTCCAT-3′	186 bp
Oct4	FOR: 5′-CTCACCCTGGGGGTTCTATT-3′ REV: 5′-CTCCAGGTTGCCTCTCACTC-3′	230 bp

## Abbreviations

TME	Tumor Microenvironment
ADO	Adenosine
MSCs	Mesenchymal stromal cells
EMT	Epithelial–mesenchymal transition
BM-	Bone marrow derived MSCs
CM	Conditioned medium
GBM	Glioblastoma
GMT	Glial–mesenchymal transition
GSCs	Glioblastoma stem cells
GAMs	Glioblastoma associated macrophages
TAFs	Glioblastoma associated fibroblasts
MMPs	Metalloproteinases
AMPK	AMP-dependent protein kinase
ERK	Extracellular regulated kinase
TGF-β	Transforming growth factor beta
